# Two distinct microbial communities revealed in the sponge *Cinachyrella*

**DOI:** 10.3389/fmicb.2014.00581

**Published:** 2014-11-04

**Authors:** Marie L. Cuvelier, Emily Blake, Rebecca Mulheron, Peter J. McCarthy, Patricia Blackwelder, Rebecca L. Vega Thurber, Jose V. Lopez

**Affiliations:** ^1^Biological Sciences Department, Florida International UniversityMiami, FL, USA; ^2^Oceanographic Center, Nova Southeastern UniversityDania Beach, FL, USA; ^3^Marine Biomedical and Biotechnology Research, Harbor Branch Oceanographic Institute, Florida Atlantic UniversityFort Pierce, FL, USA; ^4^Marine Geosciences, Rosenstiel School of Marine and Atmospheric Science, University of MiamiMiami, FL, USA; ^5^Department of Microbiology, Oregon State UniversityCorvallis, OR, USA

**Keywords:** marine sponge, symbionts, diversity, archaea, pyrosequencing, 16S rRNA, microbiome

## Abstract

Marine sponges are vital components of benthic and coral reef ecosystems, providing shelter and nutrition for many organisms. In addition, sponges act as an essential carbon and nutrient link between the pelagic and benthic environment by filtering large quantities of seawater. Many sponge species harbor a diverse microbial community (including Archaea, Bacteria and Eukaryotes), which can constitute up to 50% of the sponge biomass. Sponges of the genus *Cinachyrella* are common in Caribbean and Floridian reefs and their archaeal and bacterial microbiomes were explored here using 16S rRNA gene tag pyrosequencing. *Cinachyrella* specimens and seawater samples were collected from the same South Florida reef at two different times of year. In total, 639 OTUs (12 archaeal and 627 bacterial) belonging to 2 archaeal and 21 bacterial phyla were detected in the sponges. Based on their microbiomes, the six sponge samples formed two distinct groups, namely sponge group 1 (SG1) with lower diversity (Shannon-Weiner index: 3.73 ± 0.22) and SG2 with higher diversity (Shannon-Weiner index: 5.95 ± 0.25). Hosts' 28S rRNA gene sequences further confirmed that the sponge specimens were composed of two taxa closely related to *Cinachyrella kuekenthalli*. Both sponge groups were dominated by *Proteobacteria*, but *Alphaproteobacteria* were significantly more abundant in SG1. SG2 harbored many bacterial phyla (>1% of sequences) present in low abundance or below detection limits (<0.07%) in SG1 including: *Acidobacteria*, *Chloroflexi*, *Gemmatimonadetes*, *Nitrospirae*, PAUC34f, *Poribacteria*, and *Verrucomicrobia*. Furthermore, SG1 and SG2 only had 95 OTUs in common, representing 30.5 and 22.4% of SG1 and SG2's total OTUs, respectively. These results suggest that the sponge host may exert a pivotal influence on the nature and structure of the microbial community and may only be marginally affected by external environment parameters.

## Introduction

Sponges are one of the most primitive Metazoan life forms with fossils dating from at least 580 million years ago (Li et al., 1998; Ryan et al., 2013). Today, there are more than 8500 described extant sponge species, most of which are marine (van Soest et al., 2012). Marine sponges are ecologically important components of the benthic community due to their wide diversity and high biomass (Ilan et al., 2004; de Goeij et al., 2013). In addition, they play a key functional role linking benthic and pelagic ecosystems, as they efficiently remove particulate organic carbon from the seawater (Díaz and Rützler, 2001; Ilan et al., 2004; Webster et al., 2011). Indeed, these sessile invertebrates are able to filter considerable amounts of seawater; a 1 kg sponge can filter up to 24000 L of water per day (Vogel, 1977). Because they are efficient filter feeders, many sponges can live in nutrient-poor habitats such as tropical reefs. However, because of their feeding mode, they are also directly affected by water quality and are vulnerable to marginal environmental conditions (Webster and Blackall, 2009).

Many sponge species consistently harbor dense and diverse microbial communities including bacteria, archaea and eukaryotes (Taylor et al., 2007b). Symbionts can contribute up to 50% of the sponge biomass (Wilkinson, 1978a,b,c; Hentschel et al., 2006). Sponge-associated microorganisms include members of two archaeal lineages and >30 different bacterial and candidate phyla (Taylor et al., 2007b; Webster et al., 2008; Zhu et al., 2008; Sipkema et al., 2009; Schmitt et al., 2012). Many of these taxa form monophyletic sponge-specific clusters even though they are found in geographically and phylogenetically distinct sponge hosts (Taylor et al., 2007b; Simister et al., 2012).

Although sequencing technology has revealed much about the structural diversity of sponge associated microbiomes, relatively little is known about the specific ecological relationships and interactions among these sponge symbionts and their host (Taylor et al., 2007a; Webster and Taylor, 2012). While sponges are believed to provide a favorable environment to their symbionts, the contribution of the symbionts to the host is less well understood. However, phylogenetic inference suggests that associated bacteria and archaea are capable of a range of metabolic processes that can benefit their hosts such as ammonium-oxidation (Steger et al., 2008), nitrite-oxidation (Hentschel et al., 2002), nitrogen fixation (Wilkinson and Fay, 1979), sulfate reduction (Hoffmann et al., 2005), and photosynthesis (Wilkinson and Fay, 1979; Bayer et al., 2008; Hoffmann et al., 2009; Mohamed et al., 2010; Schläppy et al., 2010). However, it is possible that sponges and some, or all, of their microbes coexist in a more commensal or even parasitic style relationship with their hosts as opposed to a truly mutualistic one.

Further, how sponges distinguish between symbionts, food and pathogens is still unclear (Webster and Blackall, 2009). Recent studies have compared sponge microbial communities from phylogenetically distant hosts in the same location and from closely related sponges at different locations (Hentschel et al., 2002; Webster et al., 2010; Schmitt et al., 2012; Jeong et al., 2013; Montalvo et al., 2014; Kennedy et al., 2014). Thus, studies have established a “core microbial community” that would be present in many host taxa under various space and time conditions (Schmitt et al., 2012).

Here, we compare the microbial communities of different specimens of the sponge genus *Cinachyrella* collected from the same South Florida location at two different times of year. *Cinachyrella* (class *Demospongiae*), is common in coastal waters of South Florida as well as the Caribbean, with three species (*C. kuekenthali*, *C. alloclada*, and *C. apion*) present in these locations (Cárdenas et al., 2009). While *C. apion* is usually small and lives mainly near the mangrove area in shallow waters, *C. kuekenthali* and *C. alloclada* typically occur on reefs (Rützler and Smith, 1992; Cárdenas et al., 2009). However, these species are extremely difficult to visually differentiate and require careful examination of the spicules for identification at the species level (Cárdenas et al., 2009, personal observation).

Much debate currently exists concerning the identification of these species, with morphological diagnostic characters conflicting with molecular phylogenies created from marker genes. For example, using the 28S rRNA gene, *cox1* gene and a combination of the two former genes and 18S rRNA, Szitenberg et al. (2013) showed that, *Cinachyrella australiensis* contains several cryptic sympatric populations. Within the present study, we explore the microbiome of *Cinachyrella* specimens collected from the same natural environment. The purpose of the study was to describe the baseline microbial community of *Cinachyrella* in order to develop this sponge as a future experimental model. Interestingly, we discovered that based on different microbial communities, our samples formed two distinct groups of sponges, independent of the time of collection, indicating that *Cinachyrella* can harbor very distinct symbionts.

## Material and methods

### Sponge and seawater collection

*Cinachyrella* specimens were collected by SCUBA diving from the Inner Reef (as defined by Walker, 2012), Broward County, Florida, USA (N 26° 03′ 01″, W 80° 06′ 18″) at a depth of 6.1 m, on Aug 2, 2011, on Oct 24, 2011, and Feb 15, 2012, under a Florida Fish and Wildlife Conservation Commission Fishing License and a Special Activity License (-12-1372-372a). Sponges were identified as the genus *Cinachyrella* (family *Tetillidae*, Sollas, 1886; van Soest et al., 2014) given their characteristic orange to yellow color, subglobular shape and hispid surface. Water temperatures reached 30.3, 23.9, and 22.8°C in August, October and February, respectively. A total of 64 individuals were collected in total. Here, we present detailed results for six individuals consisting of three individuals on October and February (henceforth labeled as Sponge 1, 2, 3 (Sp1, Sp2, Sp3) Oct and Sponge 4, 5, 6 (Sp4, Sp5, Sp6) Feb. The other 58 individuals were subjected to various experimental conditions in aquaculture, and we provide a preliminary analysis of these samples (Supplementary Material). In-depth results of the different experiments for these samples are not shown. Individuals were cut at the base with a dive knife, placed in individual Nasco Whirl Pak bags filled with ambient seawater and brought to the surface. Samples were stored in the shade and maintained at ambient seawater temperature until transported back to the laboratory (within 2 h of collection). Surface seawater was also collected each time (one replicate in October and one replicate in February) from the dive site in 50 L carboys. These seawater samples were used to confirm that microbial communities associated with the sponge were specific to the sponges and not amplified from seawater DNA. Upon return to the laboratory, sponges were quartered with a sterile knife, frozen in liquid nitrogen, and placed at −80°C for long-term storage. Seawater (0.5 L) was filtered onto a 0.22 μm Supor filter (Pall Life Science, Ann Arbor, MI) by vacuum filtration (<10 mm Hg), the filters were frozen in liquid nitrogen, and stored at −80°C.

### DNA extraction

Approximately ¼ of a sponge was used for DNA extraction. In a sterile petri dish, the sample was defrosted and the ectoderm (darker outer layer) was immediately removed using a sterile scalpel. The endoderm was transferred to a new petri dish and 5 ml of buffer (10 mM Tris pH = 7.6, 100 mM EDTA, 20 mM NaCl) was added. The sponge endoderm was minced, mixed in buffer, and the cell suspension collected into 1.7 mL tubes. These sponge suspensions were centrifuged for 15 min at 16,000 *g* at 4°C. Supernatant was decanted and the pellets transferred and extracted using the MO BIO PowerSoil DNA isolation kit according to the manufacturer's instructions (MO BIO, Carlsbad, CA).

Seawater filters also were extracted with the MO BIO PowerSoil kit to avoid yield discrepancy between DNA extraction protocols. The filters were placed into bead tubes (provided by the kit) and cut into fine pieces using sterile dissection scissors. DNA was extracted according to the manufacturer's instructions using a 2 min bead-beating step (instead of 10 min vortexing step).

### Sponge 28S rRNA gene PCR and analysis

For molecular systematics, our methods followed those proscribed by the Porifera Tree of Life project (Thacker et al., 2013). Specifically, the 28S rRNA gene was amplified using the 28F63mod (5′- ACC CGC TGA AYT TAA GCA TAT HAN TMA G- 3′) and 28R2077sq (5′- GAG CCA ATC CTT WTC CCG ARG TT- 3′) (Thacker et al., 2013). PCR consisted of one reaction of 50 μL with: 1 μM each forward and reverse primer, 1 μL of template DNA, 2.5 mM MgCl_2_, 0.2 mM dNTPs and 1.25 unit of Taq (High Fidelity Taq, TaKARa Otsu, Shiga, Japan). Thermal cycling was initiated with denaturation at 94°C for 3 min, followed by 30 cycles of: 45 s at 94°C, 60 s at 55°C, and 72°C for 6 min and a final extension step for 10 min at 72°C. PCR products were visualized on a 1.5% agarose gel (containing Gel Red). PCR products were cloned and sequenced on an ABI 377 automated DNA sequencer at the University of Alabama, Birmingham using the primer: 28R1411 (5′-GTT GTT ACA CACTCC TTA GCG G-3′). Two samples (Sp5 Feb and Sp6 Feb) had low quality sequences and were removed from the study. The nearest relative for each sequence was determined using the NCBI BLASTn tool against the GenBank non redundant database.

### 16S rRNA gene PCR and analysis

Approximately 291 bp of the 16S rRNA gene was amplified by PCR using the universal bacterial and archaeal primers (targeting the V4 region of the gene): 515F (5′- GTGCCAGCMGCCGCGGTAA- 3′) and 806R (5′- GGACTACHVGGGTWTCTAAT- 3′) (Caporaso et al., 2011), which contained a unique barcode used to tag each PCR product. This primer set was chosen because it targets a broad range of bacterial and archaeal taxa with the exception of a few groups (Bates et al., 2011; Caporaso et al., 2011). PCR consisted of two reactions of 30 μL with (for each reaction): 1 μM each forward and reverse primer, 1 μL of template DNA, 2.5 mM MgCl_2_, 0.2 mM dNTPs and 1.25 unit of Taq (High Fidelity Taq, TaKARa Otsu, Shiga, Japan). Thermal cycling was initiated with denaturation at 94°C for 3 min, followed by 30 cycles of: 45 s at 94°C, 60 s at 50 and 72°C for 90 s and a final extension step for 10 min at 72°C. PCR products were visualized on a 1.5% agarose gel (containing Gel Red). Successful reactions (i.e., with a clear band, two reactions of 25 μL) were pooled and purified with the Agencourt AMPure kit (Beckman Coulter, Beverly, MA), using 1.8× vol. of AMPure bead slurry and eluted in 10 mM Tris pH 7.5. Each sample was quantified using PicoGreen dsDNA reagent (Invitrogen, Carlsbad, CA). Purified products were sequenced on a 454 Life Science Genome Sequencer FLX (Roche) at Advanced Genetic Technologies Center at the University of Kentucky.

Sequences were analyzed using QIIME version 1.6 (Caporaso et al., 2010b). Only sequences with a mean quality score >25 and of length >280 bp were included in the analysis. Sequences were then assigned to each barcode and denoised using the denoise_wrapper option (Reeder and Knight, 2010) in QIIME. Operational Taxonomic Units (OTU) were picked using the UCLUST method (Edgar, 2010) and sequences with ≥97% identities were considered as one OTU. A representative sequence was chosen for each OTU and the taxonomic identity of each representative was assigned (in QIIME) using the RDP Classifier (Wang et al., 2007) against the Greengene 12_10 database (McDonald et al., 2012). Chimera sequences were removed using the ChimeraSlayer option (Haas et al., 2011). Sequences were aligned (using PyNAST with default paramaters set in QIIME, Caporaso et al., 2010a) and screened with Lane mask to remove gaps and hypervariable regions (Lane, 1991). A representative phylogenetic tree was built using FastTree (Price et al., 2010) and used for further analysis in QIIME (alpha, beta diversity from weighted UniFrac, Lozupone and Knight, 2005 and principal coordinate analysis generated from the UniFrac distances). *T*-tests (Microsoft Excel) were used to compare the relative abundance of each microbial phylum present in the samples of SG1 and SG2. A P value less than 0.05 was considered statistically significant. A principal coordinate analysis generated from the weighted UniFrac distances and an analysis of similarity (ANOSIM, 999 permutations) were generated in QIIME for all the 64 sponge individuals.

## Results

### Molecular phylogenetics confirm sponges are *cinachyrella*

All the partial 28S rRNA gene sequences obtained were most similar to the single *C. kuekanthali* 28S rRNA sequence present in Genbank (KC869490.1). Two 28S rRNA gene sequences (Sp5 and Sp6) could not be included in this study because of poor quality. Sp1 Oct and Sp4 Feb displayed 97% identity to *C. kuekanthali* and Sp2 Oct and Sp3 Oct had 99% identity to the same sequence (*C. kuekanthali)*. Results showed that Sp1 Oct and Sp4 Feb were most closely related to each other (99.3% identity compared to ~97% identity to the other two samples). Similarly, Sp2 Oct and Sp3 Oct were 100% identical to each other respectively, but only ~97% identical to the other two samples (Table 1). Based on the 28S rRNA gene sequences, the samples therefore form two groups, one group including: Sp1 Oct and Sp4 Feb and another group including: Sp2 Oct and Sp3 Oct. These are similar to the two groups observed after analysis of the microbiomes (see below).

**Table 1:** **Percent identity between the 28S rRNA gene partial sequences of *Cinachyrella* samples (Sp1- 4: sponge 1- 4) collected in October 2011 (Oct) and February 2012 (Feb) from South Florida and *C. kuekenthali* (*C. kuek*.; GenBank: KC869490.1; Panama)**.

	**Sp1 Oct**	**Sp2 Oct**	**Sp3 Oct**	**Sp4 Feb**	***C. kuek***.
Sp1 Oct	100				
Sp2 Oct	97.3	100			
Sp3 Oct	97.3	100	100		
Sp4 Feb	99.3	96.9	96.9	100	
*C. kuek*	96.9	99.8	99.8	96.5	100

### *Cinachyrella* specimens harbor a diversity of unique bacteria and archaea

After quality control and chloroplast sequence removal, a total of 16,811 sequences were analyzed including 13,947 from sponges (ranging from 1185 to 3616 sequences/animal) and 2864 from seawater (ranging from 1340 to 1524 sequences/sample) (Table 2). Results indicated that *Cinachyrella* specimens harbor a diverse community of symbionts, including members of all three Domains of life (Bacteria, Archaea and Eukaryotes). Here, the analysis of the eukaryotic community is not presented. In total, 951 OTUs (measured at 97% identity) were identified among all samples (including seawater), of which 19 were archaeal and 932 were bacterial. A total of 639 OTUs (12 archaeal and 627 bacterial OTUs) were present in the sponge symbiont community, and OTU richness in the sponges was lower than the seawater except for one sample (Sp3 Oct, 341 OTUs). The seawater microbial community contained a total of 450 OTUs (10 archaeal and 440 bacterial OTUs), and OTU richness was similar in both samples (246 vs. 285 OTUs) across sampling times (Table 2).

**Table 2:** **Overview of the number of sequences, OTUs (97% identities) and diversity indices for six sponges (Sp1- 6: sponge 1- 6) and seawater (SW) samples collected in October 2011 (Oct) and February 2012 (Feb)**.

**Sample ID**	**Total #reads**	**Total OTUs^#^**	Chao1 ^*^	Observed OTUs ^*^	Shannon ^*^
**SEAWATER**					
SW Oct	1340	246 (0)	440	221	6.2
SW Feb	1524	285 (1)	510	239	6.3
**GROUP “SG1”**					
Sp1 Oct	1185	90 (2)	176	86	3.7
Sp4 Feb	2386	179 (0)	267	119	4.2
Sp5 Feb	1755	105 (1)	191	79	3.3
Sp6 Feb	3616	115 (1)	124	61	3.1
**GROUP “SG2”**					
Sp2 Oct	2254	220 (1)	289	156	5.7
Sp3 Oct	2751	341 (0)	529	203	6.2

# *Number in parentheses denotes the number of unclassified OTUs included in the total*.

* *1100 reads were subsampled to calculate diversity indices*.

### *Cinachyrella* contain distinct and canalized microbiomes compared to seawater

Rarefaction analysis demonstrated that for some samples (Seawater Oct, Seawater Feb, Sp2 Oct and Sp3 Oct), the diversity was high enough such that sequencing depth was likely not sufficient to evaluate the rarer members of the community and that further sequencing would be necessary to reveal the true diversity (Supplementary Figure 1). Yet the rarefaction analysis here confirmed that most sponge samples' microbiome was less diverse than seawater (Supplementary Figure 1). Chao1 richness estimates for sponges varied from 124 to 529 phylotypes and 440 and 510 OTUs for the seawater (*t* = −1.9, 0.05 < *P* < 0.1). Similarly, the Shannon-Wiener indices for the *Cinachyrella* samples were lower on average (3.1–6.2), but not statistically different than for the seawater (6.2 and 6.3; Table 2 *t* = −1.9, 0.05 < *P* < 0.1).

Comparatively, 21 bacterial and 2 archaeal phyla and candidate phyla were detected in the sponges vs. 27 bacterial and 2 archaeal phyla and candidate phyla in the seawater. Here, we use the term “candidate phylum” to define a phylum that can be identified from genetic sequences, but lacks cultured representatives (Hugenholtz et al., 1998). Most bacterial sequences were classified, but a small portion (2.7 ± 0.9% in sponges and 2.8 ± 0.003% in seawater samples) could not be assigned to any known phylum.

### Microbial community composition defines two *cinachyrella* taxa

**Figure 1:**
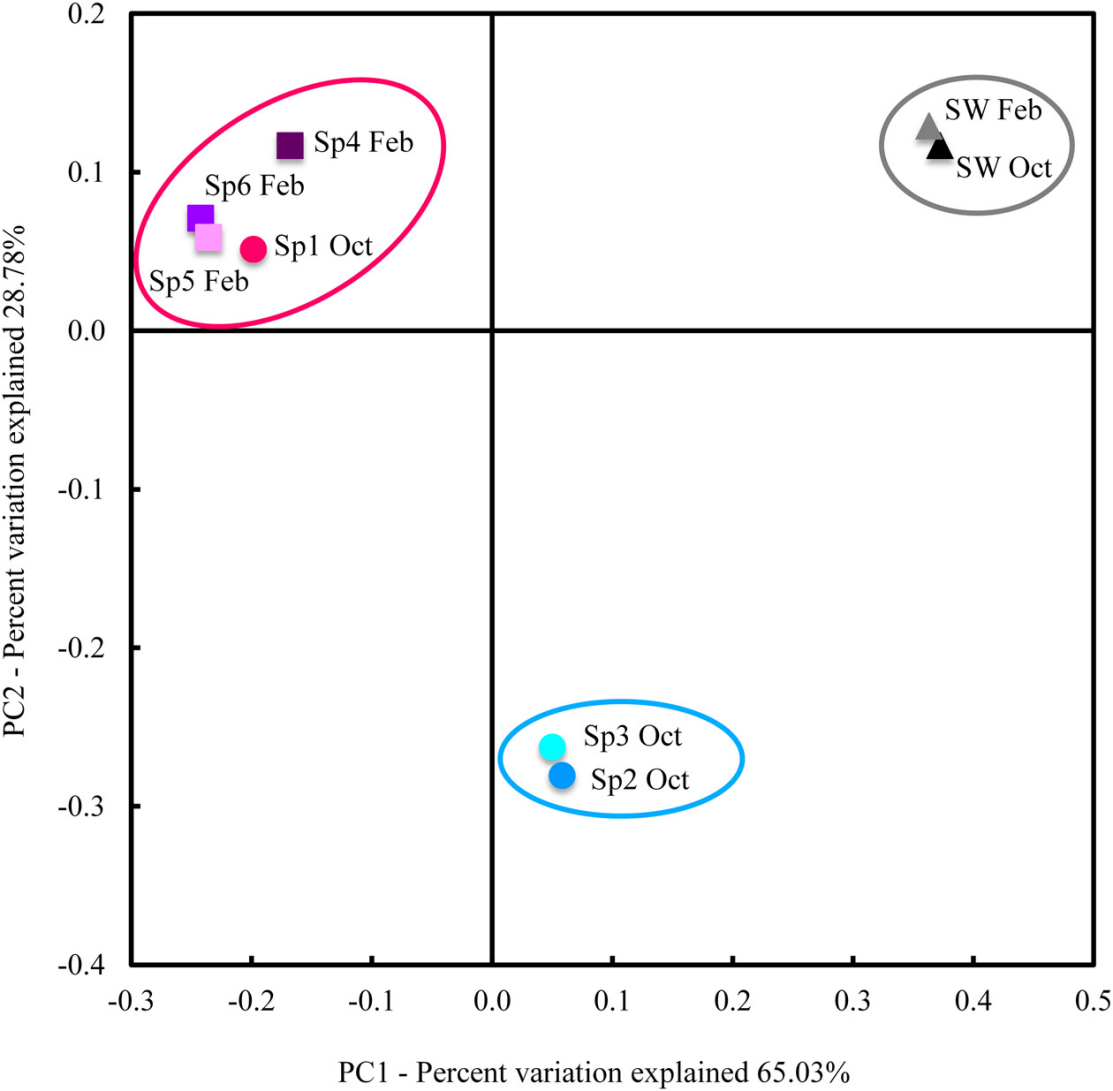
**PCoA analysis of weighted UniFrac distance**. UniFrac measures phylogenetic distances between OTUs sets within a phylogenetic tree. Here, we used weighted UniFrac, which takes into account relative abundances of OTUs (as opposed to presence/absence only). Samples formed three groups: a seawater group (samples circled in gray), SG1 (samples circled in red) and SG2 (samples circled in blue). Ovals circling samples are for visual guidance and do not represent any statistical grouping.

Both sequence taxonomy (Table 1) and PCoA analyses (Figure 1) suggest that the *Cinachyrella* specimens in this study form two distinct groups and may represent different taxa of sponge. We defined here these groups as Sponge Group 1 (SG1) and Sponge Group 2 (SG2; Figure 1). SG1 incorporates samples that spanned both seasons (Sp1 Oct, Sp4 Feb, Sp5 Feb, and Sp6 Feb) while SG2 is composed of just two samples from one season (Sp2 Oct and Sp3 Oct). In addition, the PCoA analysis for all 64 sponges samples (See Material and Methods) confirmed that Sp1-6 were split among two groups of sponges defined by their microbial communities (Supplementary Figure 2), even though 58 of these samples were placed in aquaculture under various conditions (results of experiments not shown). ANOSIM analysis (using all 64 sponge samples) confirmed that these were statistically different (*R* = 0.9926, *P* = 0.001).

The marked differences in these two groups are demonstrated by comparisons of the diversity of microbial taxa in each. SG2 samples harbored a more diverse community of microbes as measured by a mean Shannon-Wiener diversity index of 5.95 ± 0.25 (s.e.m.) compared to 3.73 ± 0.22 in the SG1 community (*t* = −6.8, *P* < 0.01; Table 2). Further, SG2 contained taxa from 21 different bacterial phyla and candidate phyla and 2 archaeal phyla; SG1 contained about half that with 12 bacterial and candidate phyla, and 2 archaeal phyla.

**Figure 2:**
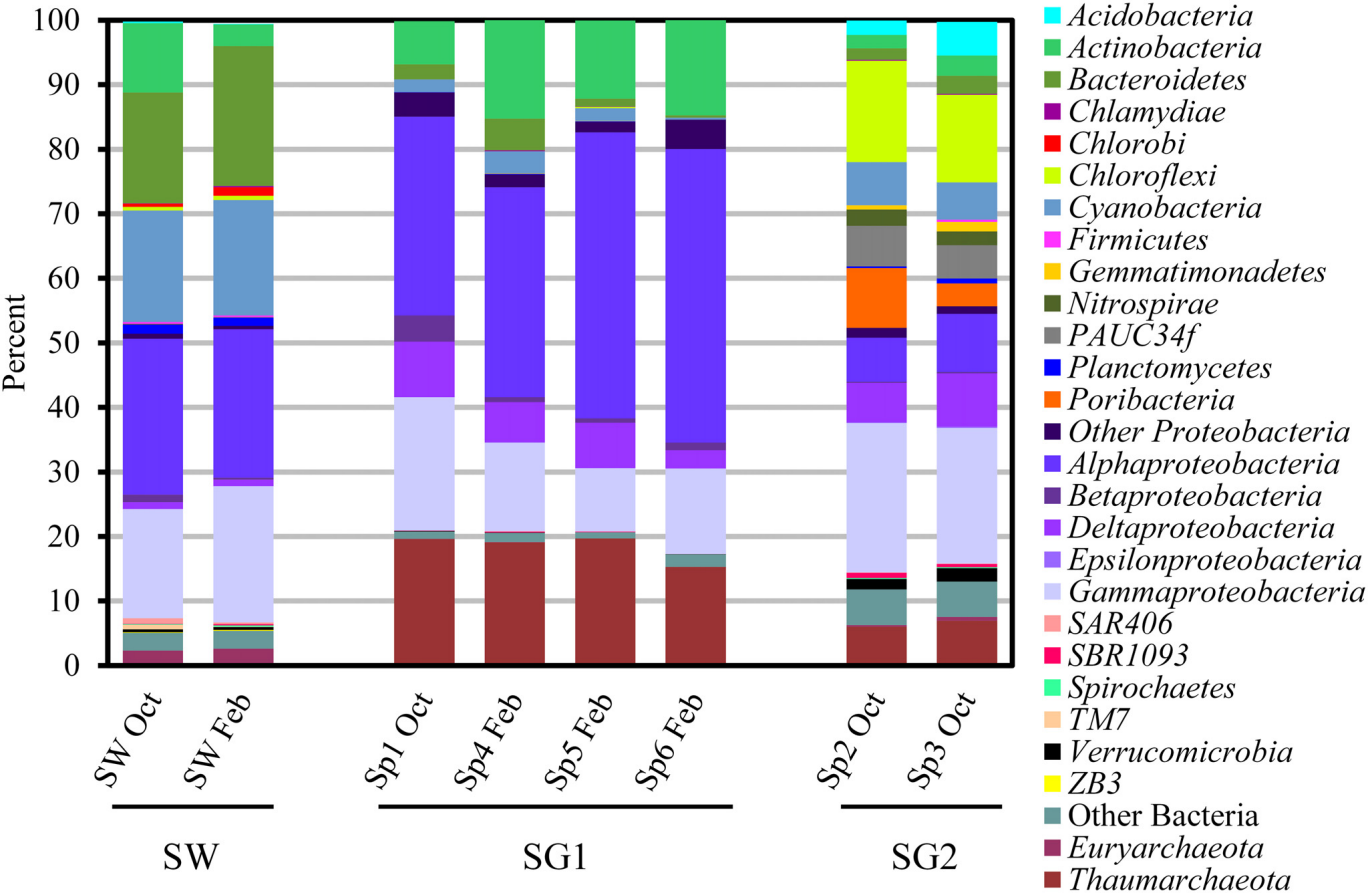
**Relative abundance of pyrosequencing reads at the phylum (or classes in the case of *Proteobacteria*) level present in six sponges (Sp1- 6: sponge 1- 6) and seawater (SW) samples collected in October 2011 (Oct) and February 2012 (Feb)**. Phyla comprised of <0.1% of sequences per sample are not shown. Based on the microbial community structure, samples were placed into two groups: sponge group 1 (SG1: Sp1 Oct, Sp4 Feb, Sp5 Feb and Sp6 Feb) and sponge group 2 (SG2: Sp2 Oct and Sp3 Oct).

Overall, both sponge groups were dominated by *Proteobacteria* (SG1: 63.5 ± 2.9%; SG2: 38.9 ± 1.0%), but *Alphaproteobacteria* were more abundant (*t* = 5.23, *P* < 0.01) in SG1 (38.3 ± 3.8%) than in SG2 (7.9 ± 0.2%). *Proteobacteria* in SG2 were dominated by the *Gammaproteobacteria* (22.1 ± 1.1%, Figure 2). *Actinobacteria* were also present in both sponge groups, but were in significantly greater numbers (*t* = 3.23, *P* < 0.05) in SG1 (12.2 ± 2.0%, Figure 2) than SG2 (2.6 ± 0.6%, Figure 2). SG2 harbored the candidate phylum *Poribacteria* (6.4 ± 2.9%) that was first discovered from sponge tissues and can be widespread in these invertebrates (Fieseler et al., 2004; Lafi et al., 2009). In contrast *Poribacteria* was below the detection limit in SG1 (*t* = −3.67, *P* < 0.05; Figure 2).

Only a few bacterial phyla or classes were not significantly different in abundance between SG1 and SG2: *Bacteroidetes* (*t* = −0.049, *P* > 0.05), *Chlamydiae* (*t* = −2.08 *P* > 0.1), *Firmicutes* (*t* = −1.63, *P* > 0.1), *Beta*- (*t* = 1.22, *P* > 0.1), *Delta*- (*t* = 0.08, *P* > 0.1), *Gammaproteobacteria* (*t* = −2.23, *P* > 0.05), and SAR406 (*t* = −0.42, *P* > 0.1, Figure 2). On the contrary, many phyla were present in SG2 at >1% (mean), but in very low abundance (<0.07% mean) or below detection limits in SG1 and included: *Acidobacteria* (*t* = −4.03, *P* < 0.02), *Chloroflexi* (*t* = −22.09, *P* < 0.001), *Gemmatimonadetes* (*t* = −4.154, *P* < 0.02), *Nitrospirae* (*t* = −18.01, *P* < 0.001), PAUC34f (*t* = −17.63, *P* < 0.001) and *Verrucomicrobia* (*t* = −11.99, *P* < 0.001, Figure 2).

In SG1, a few OTUs noticeably dominated the community and composed >10.0% of all the sequences. These included one unclassified *Alphaproteobacteria* OTU (30.0 ± 4.4%), one OTU in the *Cenarchaeaceae* family (18.3 ± 1.1%; Supplementary Figure 3), and one unclassified *Actinobacteria* OTU (11.9 ± 2.0%). In SG2, none of the OTUs represented more than 10% of all the community.

Another striking difference in the communities was the relative abundance of archaeal sequences. Archaeal sequences represented a large portion (18.5 ± 1.1%) of all the sequences recovered from SG1 samples, but only 6.9 ± 0.7% for SG2 samples (*t* = 9.23, *P* < 0.01; Figure 2). In SG1, one archaeal OTU in *Cenarchaeaceae* family (mentioned above) was dominant (99.3 ± 0.3%). In SG2, 68.2 ± 15.0% of archaeal reads also fell into one *Cenarchaeaceae* family OTU, but this OTU was different from the main one in SG1. A small proportion (5.8 ± 2.3%) of the SG2 archaeal sequences were assigned to the phylum *Thaumarchaeota*, which was almost absent (except for three sequences) from SG1 (*t* = 7.48, *P* < 0.01). These data indicate that the sponges collected in our study, while physically reminiscent, in the same genus, and from the same environment harbor distinct enough microbial communities to warrant a re-evaluation of their phylogenetic relationship.

### Seawater archaeal and bacterial communities are distinct from sponges'

In the overlying seawater, *Proteobacteria* (45.0 ± 0.9%)—and particularly *Alpha*- (23.6 ± 0.6%) and *Gamma*- (19.0 ± 2.1%)—were the most abundant taxa of bacteria. In addition, *Bacteroidetes* (19.4 ± 2.2%)*, Cyanobacteria* (17.6 ± 0.3%), and* Actinobacteria* (7.0 ± 3.7%) were the only other bacterial phyla that comprised >2% of all the reads.

The seawater-derived archaeal sequences represented 2.5 ± 0.2% of sequences and mostly belonged to the *Thaumarchaeota*, in particular the Marine Group II or Marine Group III. Marine Group II represented 89.3 ± 4.4% of all seawater archaeal sequences with a single OTU with pronounced dominance (58.5 ± 6.0%; Supplementary Figure 2).

### *Cinachyrella*'s core and variable microbial communities

**Figure 3:**
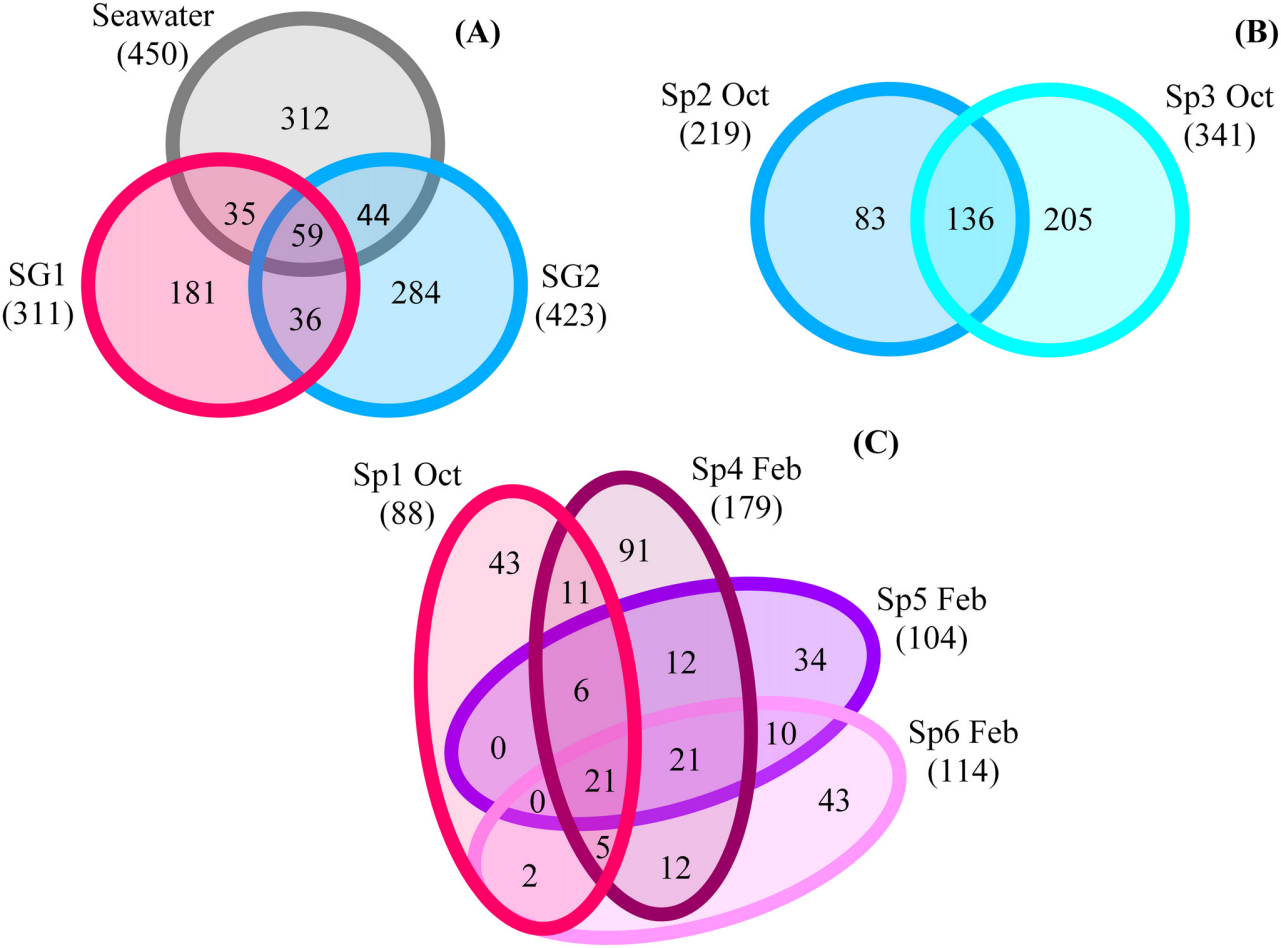
**Venn diagrams of specific and shared classified OTUs between. (A)** Sponge group 1 (SG1)-SG2-seawater (SW); **(B)** SG1 samples; **(C)** SG2 samples.

To further examine the distinct microbial communities, core and variable members of each group were compared. The numbers of common OTUs between SG1 and SG2 was relatively low, with only 95 shared OTUs representing 22.4% of the OTUs in SG2 and 30.5% in SG1. This was approximately equivalent to the numbers of OTUs the seawater shared with SG1 (94 OTUs) and SG2 (103 OTUs, Figure 3A).

Within each sponge group, 136 common OTUs were found in SG2 samples, as compared to 21 shared in the two SG1 samples (Figures 3B,C). In SG2, these common OTUs belonged to 2 archaeal and 14 bacterial phyla, with the most abundant being (≥8 shared OTUs): *Bacteroidetes*, *Chloroflexi*, *Cyanobacteria*, and *Proteobacteria*. Interestingly, the samples in SG2 also shared 12 unclassified bacterial OTUs. In SG1, the shared OTUs belonged to the *Crenarchaeota*, *Actinobacteria*, *Bacteroidetes*, *Cyanobacteria* and *Proteobacteria*. The diversity among sponge samples of the same group was similar at the class level, but not shared at the family or genus level. In most cases, many of the OTUs were present in only one of the sponge samples. Out of all the sponge samples, 83 OTUs were present in at least 50% of the samples and 23 OTUs in at least 70% of the sponge samples. Within SG1 and SG2, 107 and 424 OTUs respectively were present in at least 50% of the samples and 55 and 135 OTUs respectively were present in at least 70% of the samples. The only 11 OTUs common to all the sponge samples (i.e., the core community) were assigned to the *Proteobacteria* (*Alpha*- and *Gamma*-) as well as *Cyanobacteria*, the *Bacteroidetes* and the *Actinobacteria*.

### Microbial community functional insights

QIIME analysis of the 16S rRNA gene sequences revealed that microbes with potential contribution to the nitrogen cycle were present. SG2 samples contained OTUs belonging to the genera *Cenarchaeum* (18.4 ± 12.1% of the archaeal reads) and *Nitrosopumilus* (6.9 ± 5.9% of archaeal reads). These genera are part of the ammonia-oxidizing archaea (AOA) that oxidize ammonia to nitrite (Preston et al., 1996; Walker et al., 2010). In SG1, AOA sequences belonging to the family *Cenarchaeaceae* were also present. Bacteria involved in the second step of nitrification, the oxidation of nitrite to nitrate were present in SG2 samples. These belonged to two OTUS in the family *Nitrospiracea* (phylum *Nitrospirae*, 2.3 ± 0.2%; Supplementary Figure 3) with 98.3 ± 0.02% of these reads affiliated to one OTU. This OTU had 99% identity to sponge-derived sequences

In the *Chloroflexi* (which was almost absent from SG1), two classes were abundant in SG2: *Anaerolinae* (7.4 ± 1.6%) and SAR202 (6.4 ± 0.6%), with most OTUs in the latter class belonging to sponge-specific clusters.

The most abundant *Cyanobacteria* OTU in SG1 (1.6% ± 0.6%) and SG2 (4.7% ± 0.1%) was 100% identical to *Synechoccocus* strain WH8109. This also was the second most abundant OTU in seawater. One noteworthy finding related to *Cyanobacteria* involved the numerically dominant OTU in seawater, which was 100% identical to a UCYN-A clone, *Candidatus Atelocyanobacterium thalassa* (Thompson et al., 2012), a widespread cyanobacterium and likely a significant contributor to N_2_-fixation in marine waters (Zehr et al., 2001; Moisander et al., 2010).

In the phylum *Proteobacteria*, many OTUs were obtained that could not be further classified, but had little overlap between SG1 and SG2. In SG1, the unclassified sequences in each of the *Proteobacteria* class had one clear dominant OTU. In the *Alpha-, Beta*-, *Delta*- and *Gammaproteobacteria* sequences, this OTU encompassed 77.4 ± 4.2%, 92.1 ± 3.2%, >95.5 ± 1.4%, 55.4 ± 5.9% of the reads in each class, respectively. In contrast, in SG2, none of the unclassified OTU at the class level included more than 37.1 ± 0.2% of the sequences and a few abundant OTUs were usually present. In all sponge samples, many unclassified OTUs (at the class level) were closely related to uncultured bacteria derived from sponge tissues. In particular, the most abundant unclassified *Alpha*- and *Gammaproteobacteria* OTUs were 99 and 100% identical, respectively, to a sequence from *Cinachyra* sp. from India. Within the classified *Alphaproteobacteria*, the families *Rhodobacteraceae* and *Rhodospirillaceae* were common and diverse in both sponge groups and seawater. As expected *Pelagibacteraceae* were the most abundant *Alphaproteobacteria* in the seawater.

## Discussion

Since our field collections were confined to a relatively small portion of the reef, we did not intend or expect to collect two apparently divergent *Cinachyrella* taxa. The sponges in this study were collected as part of a broader study involving greater number of specimens used for aquaculture. Upon analysis of the all the samples, it became clear that sponges formed two groups based on their microbial communities. The sponges in aquaculture (data not shown) were subject to different conditions. We therefore decided to present here only the data from sponges collected from the reef and never kept in aquaculture. In the present study, although we have confirmed that these specimens belong to the genus *Cinachyrella*, their exact taxonomic and phylogenetic identification goes beyond the scope of this paper, as the taxonomy of this genus and family (*Tetillidae*) is still under much debate (see introduction and Szitenberg et al., 2013). However, our findings are consistent, but not totally sufficient (due to low sample number and the low 28S rRNA sequence quality of two of our six samples) to prove the idea presented by Cárdenas et al. (2012) that microbiome signatures may be useful traits to delineate some sponge taxa. Thus, additional samples and a more comprehensive histology and electron microscopy analyses of the spicules would be needed to confirm the species identity of these sponge individuals. However, given the clear differences in the microbiomes of these sponge taxa, a simple PCR diagnostic of one or more variable members of the sponges' microbiota could also be used.

Overall, our results are similar to those of Chambers et al. (2013). There, the authors showed that two sponge morphs initially assigned to the genus *Paratetilla* (Demospongiae, Tetillidae) had different microbial communities, sharing less than 43% similarity. Within each morph group, microbial community similarity varied between 65 and 94% between individuals. Using *COI* gene, the authors confirmed that one of the sponge morphs actually belonged to the genus *Cinachyrella*, “challenging the value of the morphological characters used in the classification of these genera” (Chambers et al., 2013). Similar to our results, the bacterial communities were different for the two groups, even for specimens collected from the same location.

### Diverse microbes are present in *cinachyrella*

Multiple studies have shown that marine sponges can harbor a large diversity of microbes and the microbial taxa richness present in our *Cinachyrella* tissue samples (90–341 OTUs) was within the range of other sponge species. An extensive study targeting 32 species from eight different locations worldwide revealed each sponge carried between 225 and 364 OTUs (at 97% identity) with sequence coverage similar to our study (Schmitt et al., 2012). As expected, when sequencing depth was much greater, OTU richness was higher, reaching numbers between 1099 and 2996 OTUs (95% identity) in three Pacific sponge species (Webster et al., 2010). Total taxon richness (at a higher sequencing depth) was also greater in *C. australiensis* sampled from the coast of Indonesia, in which 800 phylotypes were present (Cleary et al., 2013). In subtropical waters of Key Largo, FL, USA (close to our study site), the barrel sponge *Xestospongia muta* had Shannon diversity indices comparable to the lower range of our *Cinachyrella* samples (Montalvo and Hill, 2011). However, *Cinachyrella* contained fewer OTUs than *Axinella corrugata* (at least 1000 OTUs per specimen) collected less than a few miles away from our study site (White et al., 2012). Compared to the coral *Orbicella faveolata* (formerly *Montastraea faveolata*; Kimes et al., 2013), our sponge samples showed similar diversity, for which 943 bacterial clones contained 178 OTUs (97% similarity threshold), with Chao1 estimates of 307 ribotypes (Sunagawa et al., 2009). Similarly, the coral *O. annularis* sampled from various sites at Curaçao Island harbored 163–323 bacterial OTUs (Barott et al., 2011).

### *Cinachyrella* harbor functionally diverse microbes

A small percentage of the bacterial 16S rRNA gene fragments could not be further classified indicating that some of the bacterial diversity remains unexplored. This number was much lower than those reported for *A. corrugata* collected nearby, in which 36% of the reads obtained by amplification of the 16S rRNA gene V1-V3 regions were not assigned to any bacterial phylum (White et al., 2012). In their pyrosequencing study of *C. australiensis* and *Suberites diversicolor* microbiomes, Cleary et al. (2013) also found 34% of bacterial OTUs unclassified at the phylum level. There, the primers used targeted the V3-V4 regions while the V4 region was used for this *Cinachyrella* study.

In sponges, the dominant microbial phyla can vary with taxonomy and across geographical location or habitat. High microbial abundance (HMA) sponges usually harbor many bacterial taxa while low microbial abundance (LMA) sponges typically have one or few numerically dominant taxa and a few less abundant ones (Hentschel et al., 2003; Giles et al., 2013). In this study, SG1 samples contained few taxa with pronounced dominance, resembling LMA sponges in terms of microbial equitability, but also encompassed many other phylotypes, atypical of LMA sponges. SG2 samples clearly harbored a more diverse microbial community, similar to HMA sponges. It is important to note that the similarity of these sponge groups to HMA and LMA was inferred solely based on the structure of the microbiomes and an in-depth histological study was not performed on these samples to confirm microbial abundance.

SG2 samples contained the candidate phylum *Poribacteria*, but this taxon was below detection limits in both SG1 and seawater. This is notable because *Poribacteria* are typical members of sponge microbiomes, but have mostly been detected in HMA sponges (Hochmuth et al., 2010). This taxon can be diverse, as shown by Schmitt et al. (2012) who detected a total of 437 *Poribacteria* OTUs in the 32 sponges species studied, with up to 79 different *Poribacteria* OTUs (97% identity) per species. In our *Cinachyrella*, *Poribacteria* were only classified as two OTUs. This lower diversity related to *Poribacteria* might be distinctive of *Cinachyrella* because only four OTUs were present in *C. autraliensis* specimens from open ocean habitats in Indonesia and similar to our SG1, *Poribacteria* were undetected in specimens collected from nearby marine lakes (Cleary et al., 2013).

*Chloroflexi* also was below detection limits in SG1. This again might be typical of LMA sponges as the *Chloroflexi* were absent in LMA sponges from the Red Sea, the Caribbean Sea and the South Pacific Ocean and present in low numbers in other LMA sponges (Schmitt et al., 2011; Giles et al., 2013). In SG2, *Chloroflexi* sequences were grouped into 12 OTUs, close to the range (14–21 OTUs) Schmitt et al. (2011) reported for HMA sponges, but lower than the 502 OTUs (97% identity) retrieved from another 32 sponge species (Schmitt et al., 2012).

Giles et al. (2013) studied the microbiomes in six species of LMA sponges using clone libraries and found that the phyla *Acidobacteria*, *Chloroflexi* and *Gemmatimonadetes* were not detected. Here, SG1 samples also were missing these phyla (with the exception of three sequences of *SAR202*-*Chloroflexi* and two sequences in the *Gemmatimonadetes*). These three bacterial phyla were also missing in eight of the 13 species analyzed by Jeong et al. (2013). The other five species contained a high microbial diversity with a large proportion of *Chloroflexi* (this group was called the CF group because of the *Chloroflexi*).

We also found a large portion of unclassified *Proteobacteria* in the sponge, but not in the seawater suggesting that it was not a consequence of the analysis. In the sponges *Raspailia ramosa* and *Stelligera stuposa*, 32 and 17% of the *Proteobacteria* sequences, respectively, were unclassified as opposed to only 1% in the seawater (Jackson et al., 2012). Further exploration suggests that many of our unclassified *Proteobacteria* OTUs are sponge-specific and the presence of large clusters of sponge-specific and sponge- and coral-specific bacteria in the invertebrates have been described (Simister et al., 2012). Interestingly, our results related to *Proteobacteria* were similar to Cleary et al. (2013). In their study, *Alphaproteobacteria* were more abundant in *C. australiensis* from marine lakes than open ocean habitats. In our *Cinachyrella* samples, *Alphaproteobacteria* were significantly more abundant in the SG1 than SG2. These might again be typical of some LMA sponges as Kamke et al. (2010) also recovered a large portion of *Alphaproteobacteria* clones from LMA sponges.

*Cinachyrella* symbionts also belonged to the Archaea (6.9–18.5%), in proportions within the wide range recorded for four deep water (4–65%) and three shallow water sponges from the Red Sea (4–28%) (Lee et al., 2011; Kennedy et al., 2014). All of the archaeal sequences in *Cinachyrella* fell within two phyla: *Thaumarchaeota* and *Euryarchaeota*, with most of the archaea belonging to the *Thaumarchaeota*, which is widespread in sponges (Webster et al., 2001; Margot et al., 2002; Lee et al., 2011; Kennedy et al., 2014; Polónia et al., 2014). Archaeal reads grouped into a low number of OTUs, with a few numerically dominant ones, similar to the four species sampled by Kennedy et al. (2014), which had 70% of the *Thaumarchaeota* sequences separated in three OTUs. The phylum *Thaumarchaeota* includes AOA performing the first step of nitrification using ammonium excreted by sponges as a metabolic waste product (Jiménez and Ribes, 2007; Bayer et al., 2008; Hoffmann et al., 2009). Ammonia oxidation by archaea is believed to be widespread in marine environments (Francis et al., 2005; Könneke et al., 2005; Schleper et al., 2005) and was detected both the LMA and HMA sponges (Schläppy et al., 2010). In addition to the AOA, nitrite-oxidizing bacteria catalyzing the second step of nitrification were found in SG2. Hentschel et al. (2002) detected early on clones affiliated with nitrite-oxidizing phylum *Nitrospirae* in sponges. The proportion of this phylum varies greatly between host species, ranging from 0.6% in *X. testudinaria* from the Red Sea (Lee et al., 2011) to 24% in *Stelligera stuposa* from Irish waters (Jackson et al., 2012). Overall, in the present study, it appears that only one group of *Cinachyrella* (SG2) harbors the microbes required for both steps of nitrification.

### The two sponge groups only share a small core microbiome

Symbionts in SG1 and SG2 were very different at the OTU level with both groups only sharing a small core microbial community as seen in many sponges. For example, *C. australiensis* from open ocean habitat and marine lakes only shared 9.4% of their OTUs (Cleary et al., 2013), lower than the percentage shared between SG1 and SG2. In contrast, the sponge genus *Xestospongia* often showed exceptionally high overlap in OTUs. For example, *X. muta* (collected from Florida) and *X. testudinaria* (from Indonesia) shared 85% of the reads (=245 OTUs) between the two species (Montalvo et al., 2014). However, after surveying 32 sponge species, Schmitt et al. (2012) concluded that phylogeny of the host (i.e., how closely related sponges were) did not correlate with the bacterial composition. Similarly, host sponge phylogeny—except for the genus *Xestospongia*—did not affect the similarity of the symbionts communities in sponges from Orpheus Island (Webster et al., 2013). Nevertheless, when triplicate individuals of the same species (including *Cinachyra* sp.) were analyzed, conserved (>65% similarity) microbial communities were observed (Webster et al., 2013). This is consistent with the pyrosequencing characterization of* A. corrugata* symbiont communities in S. Florida (White et al., 2012), which showed relatively high similarities among multiple individuals and across hundreds of km. In *Cinachyrella*, the numbers of shared OTUs between SG1 samples (12–24%) and SG2 samples (39–62%) was low. Giles et al. (2013) and Schmitt et al. (2012) suggest environmental factors such as temperature, salinity or nutrient levels might impact symbionts population structures. In their study, species from tropical waters had more similar bacterial communities. This did not hold true at a smaller scale as we observed distinct communities in the two sponge groups from the same environment, independent of spatial or temporal scales.

Considering many sponges (including *Cinachyrella*) have a reduced core and large variable microbial community, it would be reasonable to assume that different OTUs perform distinct functions within the sponge. However, using a metagenomic approach, a recent study showed that taxonomically divergent sponges can harbor phylogenetically diverse symbionts with functional equivalence (Fan et al., 2012). The authors were able to show that six sponge species possess similar functional profiles distinct from the ones obtained for the seawater microbial communities (Fan et al., 2012). These findings suggest that key functions in marine sponges might be performed by different microbial taxa and a phylogenetically similar “core microbial community” may therefore not be essential to meet the sponge requirements. Moreover, perhaps the concept of a “core” microbiome, for *Porifera* at least, may have to be redefined altogether to emphasize *function* over symbiont identity. This view may not be so far fetched when considering that bacteria can often drastically change their metabolic activities through horizontal gene transfers (Costa et al., 2009).

Together with recent and ongoing molecular microbiome analyses of adjacent coastal waters and reef invertebrate hosts (unpublished), this study contributes to a growing spatio-temporal profile of microbiome dynamics in subtropical South Florida (Negandhi et al., 2010; White et al., 2012). These results also help provide a baseline characterization for *Cinachyrella*, which may be developed for further experimental studies, due to its hardiness in aquaculture, relative ease of collection and maintenance.

## Author contributions

Marie L. Cuvelier, Emily Blake, Rebecca L. Vega Thurber, Peter J. McCarthy, and Jose V. Lopez designed research; Marie L. Cuvelier, Emily Blake, and Jose V. Lopez performed sampling; Marie L. Cuvelier performed DNA extractions and 16S rRNA amplicon preparation; Emily Blake performed sponge taxonomy analysis; Rebecca Mulheron performed 28S rRNA PCR; Marie L. Cuvelier, Rebecca L. Vega Thurber, and Jose V. Lopez analyzed data; Marie L. Cuvelier, Emily Blake, Peter J. McCarthy, Patricia Blackwelder, Rebecca L. Vega Thurber, and Jose V. Lopez wrote the paper. Funding was awarded to Jose V. Lopez, Rebecca L. Vega Thurber, Peter J. McCarthy, and Patricia Blackwelder.

### Conflict of interest statement

The authors declare that the research was conducted in the absence of any commercial or financial relationships that could be construed as a potential conflict of interest.

## Supplementary material

Supplementary Figure 1 **Rarefaction curves (note: SW Feb is under the Sp6 Feb line)**.

Supplementary Figure 2 **PCoA analysis of weighted UniFrac distance**. UniFrac measure phylogenetic distances between OTUs sets within a phylogenetic tree. Here, we used weighted UniFrac, which takes into account relative abundances of OTUs (as opposed to presence/absence only). 64 sponge individuals were used in total and Sp1 through Sp6 are labeled.

Supplementary Figure 3 **Number of OTUs, abundance and classification of all the sequences present in the seawater (Oct and Feb), Sponge Group 1 (Sp1 Oct, Sp4 Feb, Sp5 Feb, Sp6 Feb) and Sponge Group 2 (Sp2 Oct and Sp3 Oct). Each OTU is classified at the lowest ranking**.

## Abbreviations

AOA: ammonia-oxidizing archaea
AOB: ammonia-oxidizing bacteria
LMA: low microbial abundance
HMA: high microbial abundance
SG1: sponge group 1
SG2: sponge group 2.
