# Supplementary figures and images for: Two distinct microbial communities revealed in the sponge Cinachyrella
Source: Front Microbiol. 2014 Nov 4;5:581. doi: 10.3389/fmicb.2014.00581 (PMC4219487; doi:10.3389/fmicb.2014.00581)

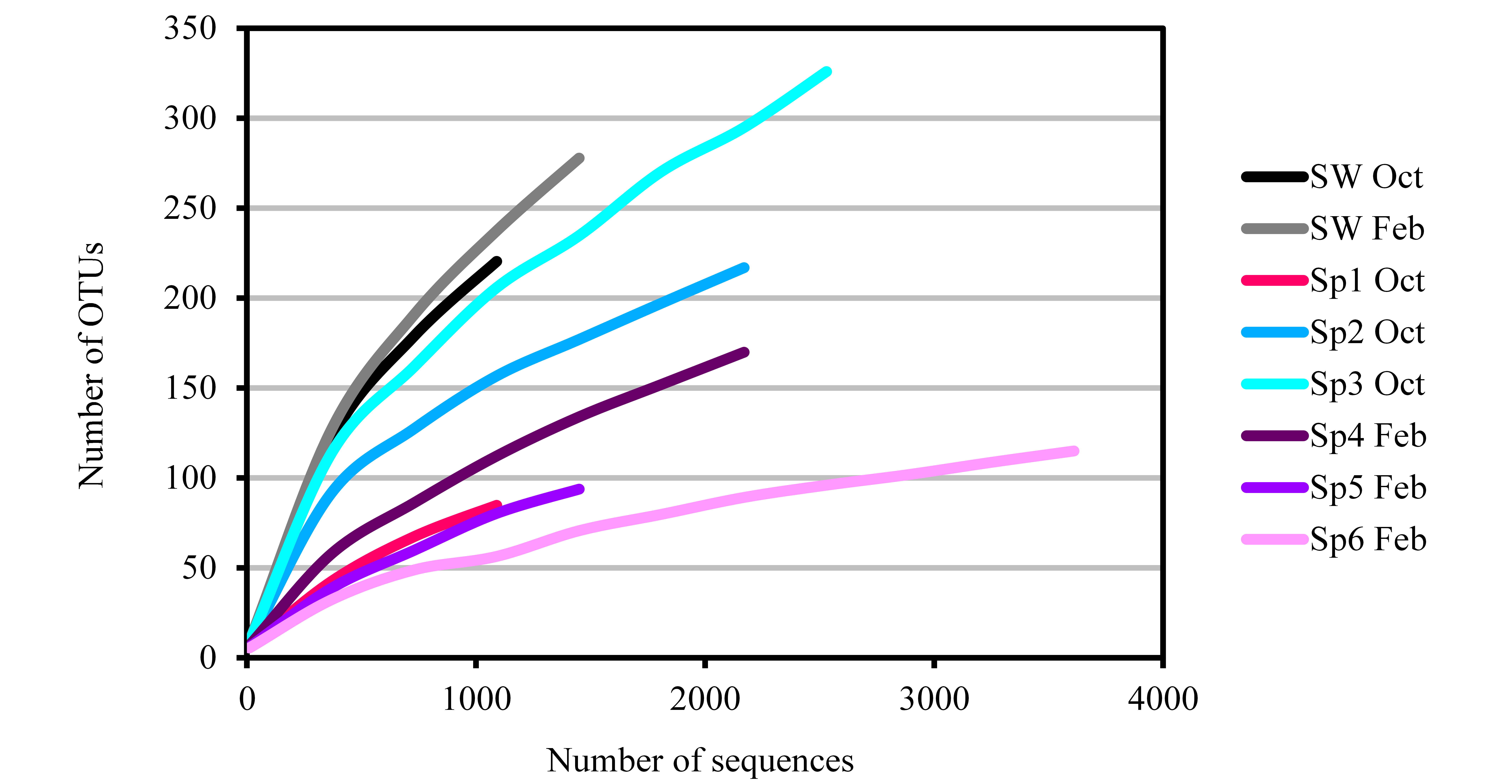

Supplement: Supplementary Figure 1 — Rarefaction curves (note: SW Feb is under the Sp6 Feb line). [file Image1.TIF]
