# Supplementary material for: Two distinct microbial communities revealed in the sponge Cinachyrella
Source: Front Microbiol. 2014 Nov 4;5:581. doi: 10.3389/fmicb.2014.00581 (PMC4219487; doi:10.3389/fmicb.2014.00581)
Supplement: Supplementary Figure 3 — Number of OTUs, abundance and classification of all the sequences present in the seawater (Oct and Feb), Sponge Group 1 (Sp1 Oct, Sp4 Feb, Sp5 Feb, Sp6 Feb) and Sponge Group 2 (Sp2 Oct and Sp3 Oct). Each OTU is classified at the lowest ranking. [file Image3.PDF]

Sup. Mat. Figure 3

# seq			# OTU
1			1
2-25			2-3
26-50			4-5
51-75			6-7
76-100			8-9
101-150			10-11
151-200			12-13
201-300			14-15
>300			>15

	Seawater		Sponge Group 1		Sponge Group 2	
	# seq	*# OTU*	# seq	*# OTU*	# seq	*# OTU*
**Archaea**					2	*1*
Thaumarchaeota						
. Cenarchaeales						
Cenarchaeaceae			1586	*3*	250	*2*
Cenarchaeum					56	*1*
Nitrosopumilus	2	*1*			20	*1*
Euryarcheota						
- Parvarchaea						
. WCHD3-30					6	*1*
. YLA114					14	*1*
- Thermoplasmata						
. E2						
Marine group II	63	*7*	3	*2*	2	*2*
Marine group III	6	*2*				
**Bacteria**	79	*44*	135	*46*	276	*66*
Acidobacteria	1	*1*				
- Acidobacteria-6					13	*2*
. BPC015	1	*1*			8	*1*
- AT-s2-57					1	*1*
- Chloracidobacteria					1	*1*
. OS-K	1	*1*				
. RB-25	1	*1*				
- Sva0725						
. Sva0725					171	*1*
Actinobacteria	1	*1*	1165	*5*	1	*1*
- Acidimicrobiia						
. Acidimicrobiales						
C111	4	*1*	1	*1*		
koll13			10	*1*	1	*1*
OCS155	84	*1*	8	*1*	9	*1*
wb1_P06	25	*2*	3	*2*	112	*4*
ZA3409c	5	*1*				
- Actinobacteria						
. Actinomycetales	2	*1*	1	*1*	6	*1*
ACK-M1	2	*2*			2	*2*
Gordoniaceae						
Gordonia	2	*1*				
Microbacteriaceae	2	*1*				
Candidatus Aquiluna						
rubra	5	*1*			2	*1*
Mycobacteriaceae						
Mycobacterium	8	*1*				
Nocardiaceae						
Rhodococcus						
ruber	13	*1*				
Nocardioidaceae	4	*2*				
Nocardioides	36	*1*				
- Nitriliruptoria						

. Nitriliruptorales						
Nitriliruptoraceae	1	*1*				
- Thermoleophilia	1	*1*				
. Gaiellales						
Bacteroidetes	35	*9*	9	*7*	5	*5*
- Bacteroidia						
. Bacteroidales	4	*2*			1	*1*
Porphyromonadaceae	2	*1*				
Prevotellaceae						
Prevotella						
copri					1	*1*
Rikenellaceae						
AF12	1	*1*				
- Flavobacteriia	171	*8*	39	*6*	14	*3*
. Flavobacteriales	16	*6*			4	*2*
Cryomorphaceae	1	*1*			6	*3*
Flavobacteriaceae	270	*24*	105	*12*	56	*10*
Aequorivita	3	*1*				
Aquimarina	1	*1*				
Flavobacterium	2	*1*				
Gramella	1	*1*				
Lutimonas	2	*1*				
Sediminicola					1	*1*
Tenacibaculum	4	*2*	2	*1*	3	*1*
Winogradskyella	1	*1*				
- Sphingobacteria						
. Sphingobacteriales	2	*2*	3	*3*	1	*1*
Amoebophilaceae			1	*1*	1	*1*
Balneolaceae	3	*1*				
Balneola	4	*1*				
SC3-56					2	*1*
Ucs1325					1	*1*
Cyclobacteriaceae	1	*1*				
Ekhidnaceae			2	*2*	3	*2*
Ekhidna					1	*1*
JTB248	2	*1*	2	*2*	1	*1*
NAC60-3	17	*1*	3	*1*		
Roseivirga	3	*1*				
Flammeovirgaceae	1	*1*	1	*1*		
A4	1	*1*				
Flammeovirga	1	*1*				
Persicobacter	1	*1*	2	*1*	3	*2*
Flexibacteraceae						
Arcicella			1	*1*		
Rhodothermaceae					7	*2*
Saprospiraceae	7	*3*	8	*5*	5	*5*
Aureispira	1	*1*				
Lewinella			1	*1*		
BRC1						
- NPL-UPA2	1	*1*				
Chlamydiae						
- Chlamydiia						
. Chlamydiales	3	*1*	3	*3*	5	*3*
Rhabdochlamydiaceae						
Candidatus Rhabdochlamydia	1	*1*				
Simkaniaceae			1	*1*	2	*2*
Chlorobi	1	*1*				
- Chlorobia						
. Chlorobiales						
Chlorobiaceae						
Prosthecochloris	19	*1*				
- Ignavibacteria						
. Ignavibacteriales	6	*1*				
IheB3-7	1	*1*				
Chloroflexi						

- Anaerolineae	4	3			175	1
. Anaerolineales						
Anaerolinaceae	1	1				
. Caldilineales						
Caldilineaceae	1	1				
. SBR1031	1	1				
A4b	2	2			204	2
SHA-31	6	3				
. SHA-20	2	1				
- SAR202			3	2	335	8
- TK17					13	1
Cyanobacteria	5	4	3	3	20	7
- 4C0d-2						
. SM1D11			1	1		
- Chloroplast	3	1	4	3	21	3
. Chlorophyta	4	4			2	1
Mamiellaceae	4	1	2	1	1	1
Ulvophyceae	1	1				
. Cryptophyta	25	2	2	1		
. Haptophyceae	22	4	4	2	5	4
. Rhodophyta			7	3	18	5
. Stramenopiles	86	8	45	6	128	7
- Nostocophycideae					3	1
- Oscillatoriophycideae						
. Chroococcales			5	2	5	4
Cyanobacteriaceae						
Cyanobacterium			1	1	1	1
Phormidiaceae						
Hydrocoleum			1	1		
Oscillatoria						
spongeliae					1	1
Spirulinaceae						
Spirulina					1	1
Xenococcaceae					1	1
Xenococcus			1	1	2	2
- Synechococcophycideae						
. Pseudanabaenales						
Pseudanabaenaceae			1	1		
Halomicronema					1	1
. Synechococcales					1	1
Synechococcaceae						
Prochlorococcus						
marinus	499	6	136	3	275	5
Deferribacteres						
- Deferribacteres						
. Deferribacterales	1	1				
Fibrobacteres						
- Fibrobacteria						
. Ucp1540	1	1				
Firmicutes	1	1			4	1
- Bacilli						
. Bacillales	1	1				
Bacillaceae	2	1				
- Clostridia						
. Clostridiales	1	1				
Clostridiaceae	2	1				
Clostridium					1	1
Fusibacter					3	1
Peptostreptococcaceae					1	1
Ruminococcaceae	1	1				
Gemmatimonadetes						
- Gemm-2	1	1	2	1	56	1
GN02						
- GKS2-174					1	1
Lentisphaerae						
- [Lentisphaeria]						

. Lentisphaerales						
Lentisphaeraceae						
Lentisphaera					1	1
. Victivallales	1	1				
. Z20	1	1				
Nitrospirae						
- Nitrospira						
. Nitrospirales						
Nitrospiraceae	1	1			115	2
NKB19	1	1				
OP3						
- PBS-25	1	1				
PAUC34f			1	1	284	3
Planctomycetes					2	2
- C6						
. D113	1	1				
- OM190						
. Agg27	1	1			2	2
. CL500-15	2	1				
- Phycisphaerae					2	2
. Phycisphaerales	12	2			7	4
- Pla3			1	1		
- Planctomycetia						
. Gemmatales						
Gemmataceae			1	1		
. Pirellulales						
Pirellulaceae	15	9	4	4	11	6
. Planctomycetales						
Planctomycetaceae						
Planctomyces	4	4				
- vadinHA49	1	1				
Poribacteria					307	2
Proteobacteria	18	11	284	20	66	17
- Alphaproteobacteria	12	8	3380	20	148	18
. Caulobacterales						
Caulobacteraceae	9	4				
Caulobacter	1	1				
. Kiloniellales	3	1			4	2
. Kordiimonadales						
Kordiimonadaceae	1	1			2	1
. Rhizobiales	8	5	3	2	1	1
Aurantimonadaceae						
Fulvimarina	1	1				
Hyphomicrobiaceae	2	1			1	1
Hyphomicrobium					1	1
Parvibaculum	3	1				
Rhodobium	3	1				
Phyllobacteriaceae						
Mesorhizobium	1	1				
. Rhodobacterales						
Hyphomonadaceae						
Hyphomonas	2	1	2	1	1	1
Maricaulis	2	1				
Oceanicaulis	4	1				
Rhodobacteraceae	180	18	35	8	42	6
Thalassobius						
mediterraneus	16	1	5	1		
. Rhodospirillales	11	1			1	1
Rhodospirillaceae	101	20	48	15	128	14
Nisaea	2	1			1	1
. Rickettsiales	75	5	34	4	35	5
Aegean_112	9	2	14	3	1	1
mitochondria					18	6
Pelagibacteraceae	215	4	38	3	8	2
. Sphingomonadales						
Erythrobacteraceae	10	2	2	1	11	2

Sphingomonadaceae	1	1				
Novosphingobium	1	1				
Sphingobium						
xenophagum	1	1				
Sphingomonas	1	1				
- Betaproteobacteria	9	3	115	3	6	2
. Burkholderiales	1	1				
Alcaligenaceae						
Achromobacter	1	1				
Comamonadaceae					2	2
Comamonas	3	1				
Variovorax					1	1
. EC94			1	1		
. Methylophilales						
Methylophilaceae						
Methylotenera	7	1	3	1		
. MWH-UniP1					1	1
. Rhodocyclales						
Rhodocyclaceae			1	1		
- Deltaproteobacteria	3	3	455	2	49	6
. Entotheonellales						
Entotheonellaceae					58	1
. Bdellovibrionales						
Bacteriovoracaceae	2	2	2	2		
Bacteriovorax	1	1	1	1		
Bdellovibrionaceae						
Bdellovibrio			7	2	168	8
. Desulfobacterales					12	1
. Desulfovibrionales						
Desulfovibrionaceae						
Desulfovibrio					1	1
cavernae	9	1				
. Myxococcales	1	1	1	1	2	2
Haliangiaceae					2	2
Nannocystaceae					4	3
OM27			1	1	1	1
. NB1-j	1	1			1	1
NB1-i					1	1
JTB38	1	1				
. PB19	2	2			1	1
. Spirobacillales			1	1		
- Sva0853	7	1	6	1	2	1
. Syntrophobacterales						
Desulfobacteraceae			1	1		
Desulfococcus			1	1	2	2
Desulfofrigus					1	1
Desulfosarcina	1	1				
Desulfotignum	2	1				
Syntrophaceae					1	1
Syntrophobacteraceae					58	3
- Epsilonproteobacteria						
. Campylobacterales						
Campylobacteraceae						
Arcobacter			1	1	7	1
- Gammaproteobacteria	73	24	1055	27	853	41
. Alteromonadales	2	2	1	1	5	2
Alteromonadaceae			1	1	1	1
Alteromonas	7	1	1	1	3	1
BD2-13	1	1			1	1
Glaciecola	4	2				
HTCC2207	1	1				
Marinobacter	10	1				
Spongiibacter			1	1	1	1
Umboniibacter			1	1		
Colwelliaceae						
Thalassomonas	2	2	6	3	5	3

Ferrimonadaceae					1	1
Ferrimonas					1	1
Idiomarinaceae						
Idiomarina	3	1				
J115	7	1				
OM60	35	2	18	1	5	1
Pseudoalteromonadaceae	1	1	1	1		
. Chromatiales	15	2				
. HTCC2188	7	1			3	1
HTCC2089	10	4	5	1	137	3
HTCC2188						
HTCC	5	2			1	1
. Legionellales			5	4		
Coxiellaceae	1	1			1	1
Coxiella			8	1		
Endoecteinascidiaceae	1	1	1	1	1	1
Francisellaceae	1	1				
Legionellaceae					1	1
. Oceanospirillales					4	1
Alcanivoracaceae	4	1				
Alcanivorax	83	2	4	2		
Endozoicimonaceae	1	1	40	1	13	1
Halomonadaceae						
Candidatus Portiera	229	7	45	6	15	4
Halomonas	5	1				
Oceanospirillaceae	3	2	3	2	4	4
Oleibacter	6	3	5	1	3	2
Oleispira			1	1		
OM182	1	1	2	1		
. Pseudomonadales			1	1		
Pseudomonadaceae	1	1	1	1		
. Thiotrichales						
Piscirickettsiaceae	1	1				
Cycloclasticus	9	1			6	1
. Vibrionales						
Vibrionaceae	4	1	13	2	8	1
. Xanthomonadales	3	2	2	2	32	1
Sinobacteraceae	6	2				
Xanthomonadaceae						
Pseudoxanthomonas						
mexicana	7	1	1	1		
SAR406						
- AB16						
. Arctic96B-7						
A714017						
SGSH944	4	4	1	1	1	1
ZA3312c	7	2	2	1	1	1
Sc-NB04	2	2				
. ZA3648c						
Aegean_185	1	1				
SBR1093						
- A712011	1	1	4	2	5	1
- EC214	1	1			27	1
- VHS-B5-50	2	1				
Spirochaetes						
- [Leptospirae]						
. [Leptospirales]						
Leptospiraceae	1	1			1	1
- GN05						
. LG048	1	1				
- Spirochaetes						
. Spirochaetales						
Spirochaetaceae	2	2				
Spirochaeta					7	1
Thermi						
- Deinococci						

. Deinococcales						
Deinococcaceae						
Deinococcus	1	*1*				
<u>Thermotogae</u>						
- Thermotogae						
. Thermotogales						
Thermotogaceae						
Geotoga	2	*1*				
<u>TM6</u>					1	*1*
- SJA-4	1	*1*			1	*1*
<u>TM7</u>	2	*2*				
- TM7-1	7	*1*				
- TM7-3	2	*1*				
<u>Verrucomicrobia</u>						
- [Pedosphaerae]	3	*2*	2	*2*	87	*1*
. Arctic97B-4	5	*2*				
- Opitutae	1	*1*				
. [Pelagicoccales]						
[Pelagicoccaceae]						
Pelagicoccus	1	*1*				
. Puniceicoccales						
Puniceicoccaceae						
Coraliomargarita					1	*1*
- Verruco-5						
. LD1-PB3					1	*1*
. R76-B128	2	*1*				
- Verrucomicrobiae						
. Verrucomicrobiales						
Verrucomicrobiaceae			1	*1*		
MSBL3	1	*1*				
Persicirhabdus					2	*1*
Prosthecobacter						
debontii					2	*1*
Rubritalea			1	*1*		
WS3						
- PRR_12						
. Sediment-1					1	*1*
<u>ZB3</u>						
- BS119	3	*3*				
